# Children’s and Adolescents’ Happiness and Family Functioning: A Systematic Literature Review

**DOI:** 10.3390/ijerph192416593

**Published:** 2022-12-10

**Authors:** Flavia Izzo, Roberto Baiocco, Jessica Pistella

**Affiliations:** Department of Developmental and Social Psychology, Faculty of Medicine and Psychology, Sapienza University of Rome, 00185 Rome, Italy

**Keywords:** happiness, subjective well-being, life satisfaction, positive affect, family functioning, developmental age, systematic review

## Abstract

Background: the present research represents the first systematic review of the literature on the relation between happiness (i.e., subjective well-being, life satisfaction, positive affect) and family functioning in families with children aged 6–18 years. Method: relevant articles were systematically searched in three scientific databases (i.e., PsycInfo, Pubmed, and Web of Science) in June 2022. The databases were searched for original articles published after 1968 with the keywords “happiness” and “family functioning.” Results: of the 2683 records recovered, 124 original articles met the eligibility criteria and were included in the review. The articles were divided according to four emergent themes: (1) family dimensions and happiness; (2) global family functioning (i.e., family functioning, and family relationships), environmental variables, and happiness; (3) parental differences; (4) longitudinal studies. Conclusions: the results of the review provide evidence for a positive relation between happiness and family functioning, across different cultures and age groups: Family dimensions (e.g., cohesion, communication) were found to strongly predict children’s and adolescents’ happiness. Future studies should investigate the differences between fathers and mothers using multi-informant and mixed methods procedures and a longitudinal research approach. The implications of the findings for children’s positive development are discussed.

## 1. Introduction

Research on children’s and adolescents’ happiness has increased in recent years [1] due to the association between happiness and improved physical and mental health [2,3]. For the present systematic review, happiness was conceptualized as a relatively stable, positive, and affective trait [4,5], with an emphasis on subjective well-being and general life satisfaction [2,6,7]. Previous studies [8,9] have suggested that family emotional bonds and positive relationships are primary sources of children’s happiness. Indeed, dimensions of family functioning have been shown to significantly predict children’s happiness, beyond the influence of peer and school settings [10]. However, to the best of our knowledge, there has been no systematic review of the relation between children’s happiness and family functioning. Thus, the present systematic literature review aimed to understand the associations between children’s and adolescents’ happiness and dimensions of family functioning.

Happiness is comprised of an affective and a cognitive component [6,11]: (a) the affective component involves high levels of pleasant emotions (e.g., joy, interest, excitement, confidence, readiness) and low levels of negative emotions (e.g., anger, fear, sadness, guilt, contempt, disgust) [12]; (b) the cognitive component represents a global assessment of quality of life, indicating the degree to which one’s essential needs, goals, and desires are satisfied [13]. These judgments are usually understood to describe overall life satisfaction, or satisfaction within a specific domain (e.g., work, family life, social life, school).

### 1.1. Family Functioning and Happiness

Previous studies have suggested that healthy family functioning is associated with children’s and adolescents’ happiness [14]. Since the 1980s, the Circumplex model [15] and the McMaster Model of Family Functioning (MMFF) [16] have promoted a new vision of the family as an open system in interaction with the environment. However, there is no single definition of family functioning in the literature. Regardless of the differing compositions of modern families, family functioning refers to effective emotional bonding between family members, the use of family rules, family communication, and the management of external events [17]. Thus, family functioning describes the dynamic interactions within a family unit and how a family fulfills its functions [18], referring to the ways in which family members interact and work together to achieve common goals and outcomes [19,20]. Various factors may influence family functioning, including family structure, socioeconomic status, life events, family relationships, and the evolutive stages of the family [19,21,22]. Although family functioning is a complex phenomenon that can be assessed in various ways [23], it generally refers to the quality of family life at a systemic level, emphasizing wellness, competence, strengths, and weaknesses [24].

Previous studies have reported that positive family functioning is associated with children’s and adolescents’ happiness [25,26,27]. In particular, research has found that family connectedness promotes well-being and parental support directly contributes to children’s happiness [28]. Furthermore, the quality of family relationships has been shown to be more important to students’ happiness than the peer group, school, or community [29].

Family cohesion and adaptability have been found to be linearly correlated with family functioning (i.e., family communication and satisfaction) [15]. Effective communication is a central feature of high family functioning [30], and research has shown that when parent–adolescent communication is good, the family is closer, more loving, and more flexible in solving problems [31]. Indeed, when defining their perceptions of well-being, adolescents frequently refer to good relationships and pleasant moments spent with family members [32].

As conflict tends to generate negative emotions, high-conflict families have been found to be associated with lower levels of happiness and life satisfaction [33]. On the other hand, family satisfaction, defined as the extent to which individuals feel satisfied with the level of perceived support from family members [34], has been shown to be associated with increased happiness and overall life satisfaction in children and adolescents [35,36,37,38]. Other studies have confirmed that a dysfunctional family relationship (e.g., low-income, family coherence, family conflict) is a risk factor for children’s and adolescents’ happiness [32,39].

### 1.2. The Present Study

Decades of research have highlighted the importance of studying children’s development within their immediate life contexts (i.e., home, school, and community) [40]. During childhood and adolescence, these contexts represent microsystems where young people spend large parts of their daily lives [2,41]. However, few studies have comprehensively examined the personal and familial factors associated with happiness as a function of developmental age. Family functioning, parent–child relationship quality, and family satisfaction have been identified as significant predictors of children’s happiness [42,43,44]. Moreover, studies have shown that happy people tend to have stronger social relationships than less happy people [45]. Research has also reported that the family plays an essential role in shaping the positive development of children and adolescents [46]. Finally, longitudinal studies have found that adolescents’ family experiences predict multiple facets of adult functioning, including physical and mental health, well-being, and academic achievement [47].

To the best of our knowledge, the present study represents the first systematic review of the literature on the relation between happiness (i.e., subjective well-being, life satisfaction, and positive affect) and family functioning during the developmental ages of 6–18 years. The importance of exploring this specific development phase derives from scientific evidence that happiness declines with increasing age [2,27,48]. Again, studies have highlighted the importance of addressing multicontextual influences on happiness, with the relevant literature strongly supporting the ecological theory, emphasizing the effects of salient life contexts [49]. In this sense, a systematic review of the literature could improve our understanding of the associations between children’s and adolescents’ happiness and dimensions of family functioning.

## 2. Methods

### 2.1. Search Strategy

The present systematic review followed the Preferred Reporting Items for Systematic Reviews and Meta-Analyses (PRISMA) guidelines [50]. Relevant articles, indexed in three scientific databases (i.e., PsycInfo, Pubmed, and Web of Science), were searched. Further studies were identified through by-hand searches of the reference lists of the included articles. The investigation was conducted in June 2022, and the search included all original research articles published post-1968.

The exact search term combinations were: ([“happi *” OR “happy” OR “positive affect *” OR “positive emotions” OR “subjective well-being” OR “subjective wellbeing” OR “well-being” OR “wellbeing” OR “life satisfaction” OR “satisfaction with life”] AND [“family funct *” OR “family conflict” OR “family cohesion” OR “family communication” OR “family flexibility” OR “family problem-solving” OR “family problem solving” OR “family satisfaction” OR “family relation*”] AND [“toddler *” OR “infant *” OR “child *” “pre-schooler *” OR “preschooler *” OR “pre-adoles *” OR “preadoles *” OR “adolesc *” OR “student *” OR “pupil *”]).

### 2.2. Study Screening Selection

Two reviewers independently selected abstracts, excluding articles that did not meet the selection criteria. Age and language filters were applied to the various databases to limit the search to studies reported in only English, French, Spanish, Italian, Portuguese, and German. Since the review focused on childhood and adolescence, studies involving participants over 18 years old were excluded. Specifically, only original research articles published in scientific journals were included in the review. Furthermore, only scientific studies using mixed or quantitative methodology were selected, while no studies involving clinical samples were included. Pure qualitative studies, books, and book chapters were excluded. No reviews examining the association between children’s and adolescents’ happiness and family functioning were found.

**Figure 1:**
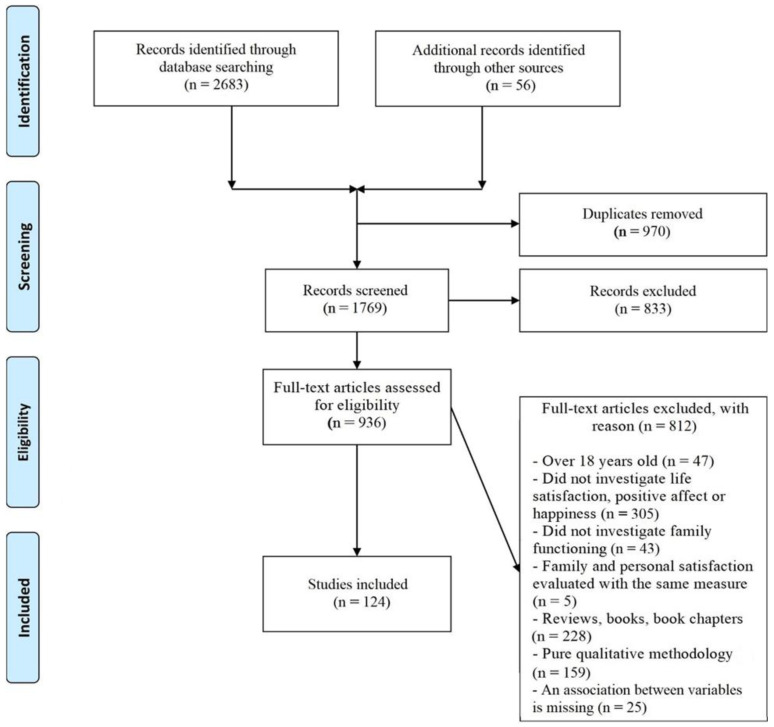
PRISMA flowchart of the study selection.

Moreover, to be considered for inclusion, studies had to assess both happiness and family functioning. Studies with a single measure evaluating the two variables as subdimensions (i.e., general life satisfaction and family satisfaction) were excluded. Only studies reporting associations between happiness and family functioning, or the effects of family functioning on children’s happiness, were included. When the results appeared vague, the researchers contacted the authors (*n* = 50) to clarify their methodology and results (*n* = 8 responded). In the absence of a response, the relevant studies were excluded. Figure 1 displays the PRISMA flowchart of the systematic review process.

### 2.3. Data Extraction

The following information was independently extracted using a structured template by two reviewers: author(s), year of publication, country, study design, participant age and gender, sample size, measures of happiness and family functioning, and main findings. Coding disagreements were resolved through discussion between the first two reviewers. The Cohen’s kappa coefficient, calculated to assess inter-rater reliability, was 0.94, reflecting very high agreement. The third author resolved any discrepancies.

## 3. Results

### 3.1. Study Characteristics

A total of 2683 scientific articles were identified (777 from PsycInfo, 662 from Pubmed, and 1244 from Web of Science), and 56 other records were added through other sources. After 970 duplicates were removed, a further 833 articles were excluded based on a review of their titles and abstracts. The remaining 936 studies were considered potentially eligible for inclusion. The full-text articles were obtained and assessed for eligibility, resulting in a final selection of 124 studies. Although the search included works published between 1968 and 2022, the present review was restricted to the years 1991–2022, because no articles published prior to 1991 met the inclusion criteria.

Regarding the study characteristics, sample sizes ranged from 74–25,906. Participant ages were also heterogeneous, though predominantly falling within the pre-adolescent and adolescent age range. With respect to school level, 18 studies examined elementary school students (i.e., aged 6–11 years) and 111 studies explored middle and high school students (i.e., aged 12–18 years). The studies were conducted in different continents: 30% in Asia (i.e., 27 in China, 1 in India, 2 in Indonesia, 3 in Israel, 3 in Korea, and 1 in Palestine), 22% in Europe (i.e., 4 in Croatia, 3 in Finland, 1 in France, 1 in Germany, 1 in Holland, 1 in Ireland, 3 in Italy, 1 in The Netherlands, 2 in Portugal, 8 in Spain, and 3 in the United Kingdom), 18% in the United States, 13% in South America (i.e., 3 in Brazil, 11 in Chile, 1 in Mexico, and 1 in Peru), and 2% in Australia. In addition, 13 articles (i.e., 11%) were cross-cultural, while 5 (i.e., 4%) were conducted in transcontinental states (i.e., 1 in Russia, 4 in Turkey). Table 1, Table 2, Table 3 and Table 4 present detailed characteristics of each of the reviewed articles, including the study design, participants, and tools.

The articles were categorized according to four emergent themes (and subthemes): (1) family dimensions and happiness; (2) global family functioning (i.e., family functioning and family relationships), environmental variables, and happiness; (3) parental differences; (4) longitudinal studies. The studies are presented in Table 1, Table 2, Table 3 and Table 4 (according to theme), and the significant findings within these four themes are synthesized in Section 3.2, Section 3.3, Section 3.4 and Section 3.5.

#### 3.1.1. Happiness Measures

The investigated studies used various measures to assess affective, cognitive, or global components of happiness. The affective component of happiness was evaluated using the Happiness Face Scale [26], Piers-Harris Children’s Concept Scale 2 (PHS) [51], Subjective Happiness Scale [52], Chinese Happiness Inventory (CHI) [53], Oxford Happiness Inventory (OHI) [54], Happiness Overall Life (HOL) [55], Happiness Taking into Account Overall Life (HTOL) [56,57], Russell’s Core Affect [58], Positive and Negative Affect Schedule (PANAS) [59], Positive and Negative Affect Scale for Children [60], Scale of Positive and Negative Affects for Adolescents (PNAA) [61], Affect Balance Scale (ABS) [62], Profile of Mood States-Adolescents (POMS-A) [63], positive affect subscales of the Profile of Mood States (POMS) [64], Personal Wellbeing Index—School Children (PWI-SC) [65], and Patients’ Well-Being Questionnaire for adolescents (PWBQ) [66].

The cognitive component of happiness was assessed using the Satisfaction with Life Scale (SWLS) [67], Students’ Life Satisfaction Scale (SLSS) [34], Cantril Ladder [68], Quality of Life Questionnaire (modified version) [69], Multidimensional Life Satisfaction Scale [70], Brief Multidimensional Students’ Life Satisfaction Scale (BMSLSS) [71], Overall Life Satisfaction (OLS) [57], Life 3 Scale [72], General Questionnaire for Adolescents [73], and Rating of Global Life Satisfaction (RGLS) [71]. Finally, the global measures of happiness were investigated using the World Health Organization—Five Well-Being Index (WHO-5 WBI) [74]), Berne Questionnaire of Subjective Well-Being/Youth form (BSW/Y) [75], Multidimensional Scale for the Measurement of Subjective Well-being of Anguas-Plata and Reyes-Lagunes (EMMBSAR) [76], and Emotional Well-Being Scale (EWS) [77].

#### 3.1.2. Family Functioning Measures

Family functioning and relationships were evaluated using nine measures, including self-report questionnaires (12 articles) and interview assessments (*n* = 1). Of the self-report measures of family functioning, the most frequently used were the Family Assessment Instrument (FAI) [78] (*n* = 7), Family Assessment Device (FAD) [23] (*n* = 6), Self-Report Family Instrument (SFI) [79] (*n* = 6), Behaviour Assessment System for Children (BASC) [80] (*n* = 2), Family Relationships Scale [81] (*n* = 2), and Family Relationship subscale of the International Survey of Children’s Well-Being (ISCWeB) [82] (*n* = 2).

Less frequently used measures (*n* = 1) included the Brief Family Function Questionnaire (BFFQ) [83], Family APGAR Index [84], Family Dynamics Measure (FDM II) [85], Family-of-Origin Scale (FOS) [86], Father/Mother Involvement Scale [87], and Relationship with Father/Mother Questionnaire (RFMQ) [88]. The only qualitative measure of family functioning was the Adolescent Interview Schedule [89], which measures the perceived family environment and the parent–adolescent relationship. Finally, some studies used specially-designed measures to investigate the quality of family relationships (e.g., [90,91]).

The investigated studies assessed specific family dimensions: (a) family cohesion and adaptability, (b) family communication and satisfaction, and (c) family conflict. Family cohesion and adaptability were evaluated using the Family Adaptability and Cohesion Evaluation Scales (FACES II, [92]; FACES III, [93]; FACES IV; [94,95]), Colorado Self-Report of Family Functioning Inventory (CSRFFI) [96], Family Environment Scale (FES) [96], and Brief Family Relationship Scale [97]. Only one study measuring family cohesion used a graphical method, applying the Pictorial Representation Index [98].

Family communication and satisfaction were assessed using the Parent-Adolescent Communication Scale [31], Attitudes and Behaviors Survey (A&B) [99], Family Satisfaction subscale of the Multidimensional Life Satisfaction Scale for Adolescents (MLSSA) [100], Family Satisfaction subscale of the Multidimensional Students’ Life Satisfaction Scale (MSLSS) [70], Family Satisfaction subscale of the Brief Multidimensional Students’ Life Satisfaction Scale (BMSLSS) [71], Satisfaction with Family Life Scale (SWFLS; Based on SWLS [67]), Satisfaction with Family Relationships (adaptation of a scale proposed by Cantril Ladder [68]), Satisfaction with Family subscale of the General Domain Satisfaction Index [101], Satisfaction with Family Life (SWFaL) [102], Family Life Satisfaction Scale (FLSS) [103], Satisfaction with Different Life Domains [104], General Family Satisfaction subscale of the Quality of Family Interaction Scale [105], and the Adolescent Interview Schedule (with the latter representing the only qualitative measure) [89].

Finally, family conflict was investigated using the Father-Adolescent Conflict Scale (FACS), Mother-Adolescent Conflict Scale (MACS) [106], Family Conflicts Scale [107], Aversive Parent-Child Interactions subscale of the Youth Everyday Social Interactions and Mood measure [108], Network of Relationships Inventory (NRI) [109], and Family Conflict subscale of the Brief Family Relationship Scale [97]. Only one study measured daily family conflict by adapting items from the Family Environment Scale [96].

### 3.2. Family Dimensions Predicting Happiness

Regarding the first theme (*n* = 91), family dimensions (i.e., cohesion and communication) were found to strongly predict children’s and adolescents’ levels of happiness. Three interconnected subdimensions characterized this theme: family cohesion and adaptability, family satisfaction and communication, and family conflict (Table 1).

#### 3.2.1. Family Cohesion and Adaptability

In the selected studies (*n* = 21), family cohesion—reflecting the strength of the family bond—was positively correlated with both the affective (i.e., positive affect and emotions) and the cognitive components (i.e., life satisfaction) of children’s and adolescents’ happiness [77,110,111,112]. Adolescents from families with higher cohesion reported a more positive mood and a higher level of happiness [111,113]. The affective component of happiness was positively correlated with family cohesion and closeness [25,114]. Feeling close to family members, doing things with family members, and sharing interests and hobbies with family members were also associated with happiness, especially in boys [25].

Children’s and adolescents’ happiness was positively correlated with family cohesion and intimacy [7,28,44,115,116,117,118,119,120]. Therefore, children who perceived a less cohesive atmosphere at home reported lower life satisfaction and higher negative affect [121], which precipitated negative thoughts towards people and events (i.e., hostility). Therefore, increased life satisfaction and low negative affect might help children to cope with adverse events [111]. In addition, Song et al. (2018) [44] found that self-esteem mediated the relationship between family cohesion and life satisfaction.

Happiness had a significantly positive correlation with family adaptability [20]—defined as the quality and expression of leadership and organization, role relationships, and rules and negotiations within the family [95]—from the perspectives of both children and parents [27]. Again, adolescents’ perceptions of family flexibility were positively associated with their happiness [122,123]. Although most studies reported that cohesion and flexibility were correlated with higher levels of happiness in children, Verrastro et al. (2020) [27] found that family variables were not significantly predictive of children’s happiness.

#### 3.2.2. Family Conflict

The examined studies highlighted that parent–child conflict (*n* = 17) strongly negatively predicted children’s and adolescents’ positive affect [77,124,125] and perceived happiness [126]. Adolescents felt less happy and satisfied on days of intense conflict with parents [113], and adequate parental warmth moderated and decreased the negative effect on children’s happiness and well-being [124]. Furthermore, parent–adolescent conflict was associated with low life satisfaction of children and adolescents [33,46,89,114,127,128,129,130], from the perspectives of both parents and children [131]. Even in late adolescence, happiness negatively correlated with family conflict before college [132].

Family conflict directly affected emotional happiness (i.e., life satisfaction and positive emotions) [77,127,133] during late adolescence. Indeed, one study found that satisfaction with life buffered the harmful effects of family conflict among undergraduate students [132]. However, other studies did not reveal a statistically-significant correlation between children’s happiness and parent–child conflict [33,134].

Adolescent gender moderated between- and within-family (i.e., daily cohesion and conflict) effects on mood, and the interaction between daily conflict and adolescent gender was significantly correlated with positive mood. One study found that, relative to girls, boys reported significantly lower levels of happiness in the context of family conflict [113]. However, another study found no gender differences among adolescents in the association between parent–adolescent conflict and adolescent psychological well-being [129].

#### 3.2.3. Family Communication and Satisfaction

In the selected studies (*n* = 13), mother–adolescent and father–adolescent communication were positively associated with both the affective component (i.e., positive affect) and the cognitive component (i.e., life satisfaction) of adolescents’ happiness [30,135]. Children’s happiness and positive affect positively correlated with family communication [25], from both the children’s and parents’ perspectives [27]. Therefore, having family members who expressed their opinions and talked about their feelings was associated with positive affect [25].

Children’s and adolescents’ life satisfaction [20,136,137] and emotional well-being (i.e., happiness, positive affect, and life satisfaction) [30] correlated positively with family communication. Specifically, adolescents’ life satisfaction was positively associated with communicative openness with their father and mother [138] and negatively with offensive and avoidant communication with their parents [114,139,140]. Some research reported that positive (i.e., accessible, comprehensive, and satisfying) family communication significantly predicted life satisfaction [138,141]. Verrastro et al. (2020) [27] found an interaction between children’s gender and family communication, suggesting that, among female participants, having a family that practiced good communication was more strongly associated with higher levels of happiness.

Moreover, studies found positive correlations between family satisfaction (*n* = 47) and happiness [142,143,144], identifying satisfaction with family life as the strongest predictor of overall life satisfaction, from childhood to adolescence [3,29,35,42,145,146]. In particular, family satisfaction correlated positively with both the affective component (i.e., positive affect and positive emotions) and the cognitive component (i.e., life satisfaction) of happiness [36,37,71,147,148,149,150]. Furthermore, family life satisfaction was positively associated with children’s positive affect [148,151,152,153] and happiness [38,126], from the perspectives of both children [1,154,155,156,157,158,159,160,161,162,163,164,165,166,167] and parents [27,73,168,169]. However, one study reported a non-significant positive correlation between happiness and family satisfaction [38].

The relation between family satisfaction and life satisfaction may be bidirectional. Indeed, one study showed that positive affect predicted high school students’ satisfaction with family life [151]. On the other hand, other studies identified family satisfaction as a significant predictor of life satisfaction [170,171,172,173]. For instance, some authors [36,149] found that high satisfaction with family life was related to a greater frequency and intensity of affective experiences of love, affection, joy, and happiness [174].

**Table 1:** Sample Characteristics and Methods of Assessment of the Reviewed Studies Investigating Family Dimensions and Happiness (*n* = 91).

	Child Characteristics			Happiness Measure		Family Measure			
Author (Year), Country	*N*	Age	% Male	Method	Measure	Method	Measure	Res. Design	Pub
Alcantara et al. (2017) [35], Brazil	910	Range 10–13 (*M* = 11.90)	47.9	S	OLS SLSS	S	SDDC	C	Pub
Bahrassa et al. (2011) [132], United States	82	Range 17–19 (*M* = 18.5)	43.9	S	SWLS	S	FCS	C	Pub
Bakalım & Taşdelen-Karçkay (2015) [151], Turkey	456	Range 14–18	47.1	S	PANAS	S	FLSS	C	Pub
Bedin & Sarriera (2015) [147], Brazil	543	Range 12–16 (*M* = 14.1)	31.7	S	HOL OLS SWLS	S	BMSLSS	C	Pub *
Bennefield (2018) [25], United States	10,148	Range 13–17 (*M* = 15.2)	48.9	S	PAS	S	FCQ FCLQ	C	Pub
Bernal et al. (2011) [36], Mexico	580	Range 15–19 (*M* = 16.45)	49.0	S	EMMBSAR SWLS	S	SWFLS	C	Pub
Bradley & Corwyn (2004) [33], United States	310	Range 15–19 (*M* = 12.24)	46.5	S	QLQ	S	FCC	C	Pub
Braithwaite & Devine (1993) [115], Australia	112	Range 14–21 (*M* = 16.62)	53.0	S	L3S	G	PRI	C	Pub
						S	PCI		
Cacioppo et al. (2013) [136], Italy	255	Range 15–17 (*M* = 15.98)	40.8	S	MSLSS	S	FAD	C	Pub
Carrascosa et al. (2018) [139], Spain	672	Range 12–19 (*M* = 14.45)	51.2	S	SWLS	S	PACS	C	Pub
Casas et al. (2007) [168], Spain (1999 sample)	1634	Range 12–16 (*M* = 14.12)	48.5	S	OLS	S	LDS	C	Pub
Casas et al. (2007) [168], Spain (2003 sample)	1618	Range 12–16 (*M* = 13.97)	46.9	S	OLS	S	LDS	C	Pub
Casas et al. (2013) [101], Spain	5937	Range 11–14	ns	S	OLS SLSS	S	GDSI	C	Pub
Casas et al. (2015) [154], Spain, Brazil, and Chile	5316	Range 12–16 (*M* = 13.59)	44.2	S	OLS	S	BMSLSS	N	Pub
Cava et al. (2014) [140], Spain	1795	Range 11–18 (*M* = 14.2)	52.0	S	SWLS	S	PACS	C	Pub
Caycho-Rodríguez et al. (2018) [142], Peru	804	Range 11–18 (*M* = 13.5)	53.0	S	WHO-5 WBI	S	SWFLS	V	Pub
Cruz & Piña-Watson (2017) [127], United States	524	Range 14–20 (*M* = 16.23)	46.9	S	BMSLSS	S	FCS	C	Pub
da Costa & Neto (2019) [155], Portugal	252	Range 15–19 (*M* = 16.87)	52.0	S	SWLS	S	SWFLS	V	Pub
Dost-Gözkan (2021) [116], Turkey	1097	Range 14–16 (*M* = 15.12)	38.4	S	MLSS	S	FES	C	Pub
Ercegovac et al. (2021) [156], Croatia	481	Range 10–17 (*M* = 12.45)	37.4	S	OLS	S	FSS	C	Pub
Estévez López et al. (2018) [114], Spain	1510	Range 12–17 (*M* = 13.4)	52.0	S	SWLS	S	PACS FES	C	Pub *
Fosco & Lydon-Staley (2020) [113], United States	151	Range 13–16 (*M* = 14.60)	38.4	S	POMS SWLS	S	FES	C	Pub
Froh et al. (2009) [148], United States	154	Range 11–13 (*M* = 12.14)	ns	S	OLS PNA	S	BMSLSS	C	Pub
Gao & Potwarka (2021) [110], China	675	Range 12–15	47.3	S	SLSS PANAS	S	FACES II	L	Pub
Galarce Muñoz et al. (2020) [152], Chile (students without disabilities)	70	Range 14–19 (*M* = 16.6)	54.3	S	PANAS	S	MSLSS	C	Pub *
Galarce Muñoz et al. (2020) [152], Chile (students with motor disabilities)	18	Range 14–19 (*M* = 15.7)	44.4	S	PANAS	S	MSLSS	C	Pub *
Galarce Muñoz et al. (2020) [152], Chile (hearing-impaired students)	17	Range 14–19 (*M* = 15.5)	76.5	S	PANAS	S	MSLSS	C	Pub *
Galarce Muñoz et al. (2020) [152], Chile (visually impaired students)	15	Range 14–19 (*M* = 16.1)	46.7	S	PANAS	S	MSLSS	C	Pub *
Gil da Silva & Dell’Aglio (2018) [153], Brazil	426	Range 12–18 (*M* = 14.9)	38.0	S	PNAA	S	MLSSA	C	Pub *
Gomez (2011) [149], United States	158	Range 11–15 (*M* = 13.49)	55.0	S	PANAS SWLS	S	MSLSS	C	Pub
Gómez et al. (2019) [1], Chile	1392	Range 10–13 (*M* = 11.5)	54.2	S	SLSS	S	GDSI	C	Pub
González-Carrasco et al. (2017) [174], Spain	970	Range 9–16 (*M* = 12.02)	44.1	S	HTOL OLS RCA	S	SDLD	F	Pub
Gross-Manos et al. (2015) [170], Israel	1081	Range 11–13 (*M* = 11.49)	51.5	S	HLTW OLS SLSS	S	BMSLSS	C	Pub
Hamama & Arazi (2012) [111], Israel	111	Range 9–13 (*M* = 11.8)	50.5	S	PANAS SLSS	S	FACES III	C	Pub
Huebner (1991a) [29], United States	79	Range 10–13 (*M* = 11.45)	63.0	S	SLSS	S	FSD	C	Pub
Ingelmo & Litago (2018) [145], Spain	1409	Range 11–18 (*M* = 14.4)	49.6	S	CL	S	SWFR	C	Pub
Irmak & Kuruüzüm (2009) [157], Turkey	959	Range 11–16 (*M* = 14.35)	50.0	S	SWLS	S	MSLSS	V	Pub
Jackson et al. (1998) [30], Holland	660	Range 13–15 (*M* = 13.5)	46.4	S	ABS CL	S	PACS	C	Pub
Jhang (2021) [175], China (Time 1)	1273	Range 12–15 (*M* = 13.55)	49.0	S	SWLS	S	FACES III	L	Pub
Jhang (2021) [175], China (Time 2)	1028	Range 14–17	ns	S	SWLS	S	FACES III	L	Pub
Jiménez et al. (2009) [138], Spain	565	Range 11–18 (*M* = 13.6)	51.0	S	SWLS	S	PACS	C	Pub
Jiménez et al. (2014) [176], Spain (Time 1)	1319	Range 12–16 (*M* = 13.5)	46.0	S	SWLS	S	PACS	L	Pub
Jiménez et al. (2014) [176], Spain (Time 2)	554	Range 12–16 (*M* = 13.7)	46.0	S	SWLS	S	PACS	L	Pub
Kaye-Tzadok et al. (2017) [171], 16 countries	5000	12-year-old children	46.2	S	SLSS	S	SWF	C	Pub
Khurana (2011) [126], India	400	Range 16–18	50.0	S	PHAS	S	MSLSS PCS	C	Pub
Kim & Main (2017) [143], South Korea and United Kingdom	3743	Range 11–12 (*M* = 12.0)	42.0	S	SLSS	S	SWF	N	Pub
Koster et al. (2018) [133], The Netherlands	255	Range 15–19 (*M* = 16.27)	57.0	S	SWLS	S	NRI	C	Pub
Leto et al. (2019) [7], Russia	424	Range 7–10 (*M* = 9.1)	49.0	S	SLSS	S	FAD	C	Pub
Lietz et al. (2020) [112], Australia	5440	Range 8–15	48.1	S	SLSS	S	ISCWeB	C	Pub
Lin & Yi (2019) [117], China	2690	Range 13–17 (*M* = 13.3)	51.2	S	LS	S	FACES III	L	Pub
Ljubetić & Reić Ercegovac (2020) [73], Croatia	101	Range 10–17 (*M* = 15.4)	31.7	S	GQA	S	QFIS	C	Pub
Mallette et al. (2021) [122], United States	207	Range 11–18	ns	S	PWI-SC	S	FACES IV	C	Pub
Manzi et al. (2006) [118], Italy and United Kingdom	223	Range 17–21 (*M* = 18.9)	49.3	S	SWLS	S	CSRFFI	N	Pub
Merkaš & Brajša-Žganec (2011) [119], Croatia	298	Range 10–15 (*M* = 12.7)	43.0	S	BMSLSS	S	CSRFFI	C	Pub
Migliorini et al. (2019) [159], Italy	1145	Range 7–10 (*M* = 8.21)	49.9	S	OLS SLSS	S	BMSLSS	C	Pub
Moore et al. (2018) [135], United Kingdom	9055	Range 11–16 (*M* = 13.7)	50.6	S	SWB	S	FCSFR	C	Pub
Moreno-Maldonado et al. (2020) [158], Portugal and Spain	21,081	Range 11–16	50.2	S	CL	S	SWFR	N	Pub
Orejudo et al. (2021) [172], Mexico, Peru, and Spain (Mexico sample)	645	Range 12–18 (*M* = 14.69)	72.6	S	LSD	S	QFR	N	Pub
Orejudo et al. (2021) [172], Mexico, Peru, and Spain (Peru sample)	1331	Range 12–18 (*M* = 14.35)	37.6	S	LSD	S	QFR	N	Pub
Orejudo et al. (2021) [172], Mexico, Peru, and Spain (Spain sample)	791	Range 12–18 (*M* = 14.45)	41.0	S	LSD	S	QFR	N	Pub
Park & Huebner (2005) [3], Korea and United States (Korea sample)	472	Range 12–17 (*M* = 15.22)	51.0	S	SLSS	S	MSLSS	N	Pub
Park & Huebner (2005) [3], Korea and United States (United States sample)	543	Range 12–17 (*M* = 14.89)	46.0	S	SLSS	S	MSLSS	N	Pub
Park (2005) [146], Korea (elementary students sample)	247	Range 9–11 (*M* = 10.7)	47.0	S	SLSS	S	MSLSS	C	Pub
Park (2005) [146], Korea (middle school student sample)	231	Range 12–14 (*M* = 13.8)	48.0	S	SLSS	S	MSLSS	C	Pub
Park (2005) [146], Korea (high school student sample)	258	Range 15–17 (*M* = 16.5)	49.0	S	SLSS	S	MSLSS	C	Pub
Park et al. (2005) [137], South Korea	501	Range 14–16	54.1	S	SWLS	S	PACS	C	Pub
Raboteg-Šarić et al. (2009) [28], Croatia	2823	Range 14–18 (*M* = 16.86)	45.5	S	GSL	S	FES	C	Pub
Rees (2017) [42], eight European countries	9156	Aged around 12 years old	ns	S	SLSS	S	BMSLSS	N	Pub
Rhatigan (2002) [123], United States	189	Range 11–14	ns	S	SWLS	S	FACES II	C	Pub
Rodríguez-Rivas et al. (2021) [128], Chile	287	Range 15–18 (*M* = 15.95)	60.3	S	SLSS	S	FC	C	Pub
Salewski (2003) [121], Germany	30	Range 14–19 (*M* = 17.2)	56.6	S	PWBQ	S	FACES II	C	Pub
Sastre & Ferrière (2000) [144], France	100	Range 12–19	50.0	S	SWLS	S	SWFR	C	Pub
Schnettler et al. (2017) [169], Chile	300	Range 10–17 (*M* = 13.2)	51.0	S	SWLS	P/S	SWFaL	C	Pub
Schnettler et al. (2018a) [160], Chile	300	Range 10–17 (*M* = 13.2)	51.3	S	SWLS	P/S	SWFaL	C	Pub *
Schnettler et al. (2018b) [161], Chile	340	Range 10–17 (*M* = 13.2)	ns	S	SWLS	P/S	SWFaL	C	Pub *
Schnettler et al. (2018c) [162], Chile	470	Range 10–17 (*M* = 13.3)	52.3	S	SWLS	S	SWFaL	C	Pub
Schnettler et al. (2018d) [163], Chile	303	Range 10–17 (*M* = 13.3)	48.5	S	SWLS	S	SWFaL	C	Pub
Schnettler et al. (2020) [21], Chile	473	Range 10–17 (*M* = 13.3)	48.2	S	SWLS	S	SWFaL	C	Pub
Schnettler et al. (2021) [164], Chile	470	Range 10–17 (*M* = 13.3)	47.7	S	SWLS	S	SWFaL	C	Pub
Schnettler et al. (2022) [165], Chile	303	Range 10–17 (*M* = 13.3)	48.5	S	SWLS	S	SWFaL	C	Pub *
Seligson et al. (2003) [71], United States	221	Range 11–14 (*M* = 12.33)	58.0	S	BMSLSS PANAS RGLS SLSS	S	MSLSS	V	Pub
Seligson et al. (2005) [150], United States	518	Range 8–11 (*M* = 9.34)	46.7	S	PANAS RGLS SLSS	S	BMSLSS	C	Pub
Shek (1997a) [46], China	365	Range 12–16	80.5	S	SWLS	S	F/MACS	C	Pub
Shek (1997c) [131], China	429	Range 12–16 (*M* = 13.0)	50.6	S	SWLS	P/S	F/MACS	D	Pub
Shek (1998b) [129], China (Time 1)	429	Range 12–16 (*M* = 13.0)	50.6	S	SWLS	P/S	F/MACS	L	Pub
Shek (1998b) [129], China (Time 2)	378	Range 13–17 (*M* = 14.0)	ns	S	SWLS	P/S	F/MACS	L	Pub
Shek (1998c) [89], China (Time 1)	429	Range 12–16 (*M* = 13.0)	50.6	S	SWLS	S	F/MACS	L	Pub
						I	AIS		
Shek (1998c) [89], China (Time 2)	378	Range 13–17 (*M* = 14.0)	ns	S	SWLS	S	F/MACS	L	Pub
						I	AIS		
Shek (2002d) [177], China	229	Range 12–16	53.3	S	SWLS	S	F/MACS	D	Pub
Shek et al. (2001) [130], China	1519	Range 11–18 (*M* = 13.5)	49.9	S	SWLS	S	F/MACS	C	Pub
Silva et al. (2020) [124], United States	120	Range 13–15 (*M* = 14.36)	39.0	S	POMS	S	YESIMM	C	Pub
Soares et al. (2019) [141], Portugal	503	Range 13–19 (*M* = 15.92)	37.0	S	SWLS	S	A&B	C	Pub
Song et al. (2018) [44], China	428	Range 11–16 (*M* = 13.16)	65.0	S	SLSS	S	FACES II	C	Pub
Sun et al. (2015) [120], China	1708	Range 14–18 (*M* = 15.03)	45.2	S	SLSS	S	FACES II	C	Pub
Taşdelen-Karçkay (2016) [173], Turkey	436	Range 14–19 (*M* = 16.35)	44.0	S	SWLS	S	FLSS	V	Pub
Tian et al. (2015) [166], China	1904	Range 9–14 (*M* = 11.25)	52.0	S	SLSS	S	BMSLSS	V	Pub
Vera et al. (2012) [37], United States	168	Range 12–15 (*M* = 13.5)	55.0	S	PANAS SWLS	S	MSLSS	C	Pub
Veronese et al. (2012) [38], Palestine	74	Range 7–15 (*M* = 10.80)	58.0	G	HFS	S	MSLSS	C	Pub
				S	PANAS				
Verrastro et al. (2020) [27], Italy	1549	Range 7–14 (*M* = 11.1)	47.0	G	HFS	S	FACES IV	C	Pub
				S	PHS				
Wang et al. (2021) [125], United States	447	Range 12–18 (*M* = 15.09)	39.1	S	PANAS	S	NRI	C	Pub
Weber & Huebner (2015) [167], United States	344	Range 11–14 (*M* = 12.23)	45.1	S	SLSS	S	MSLSS	C	Pub
Yuan et al. (2019) [20], China	703	Range 10–13 (*M* = 12.5)	54.9	S	SLSS	S	PACS FACES II	C	Pub
Yun & Choi (2018) [77], Korea	527	Range 10–12 (*M* = 11.42)	54.3	S	EWBS	S	BFRS	C	Pub
Zhao et al. (2015) [178], China (Father migrating group)	145	Range 10–17 (*M* = 13.9)	60.0	S	SWLS	S	FACES II	C	Pub
Zhao et al. (2015) [178], China (two-parent migrating sample)	96	Range 10–17 (*M* = 13.9)	55.2	S	SWLS	S	FACES II	C	Pub

Note. Happiness method: G = graphical assessment; S = self-report questionnaire. Happiness measure: ABS = Affect Balance Scale; PWBQ = Patients’ Well-Being Questionnaire for adolescents; BMSLSS = Brief Multidimensional Students’ Life Satisfaction Scale; CL = Cantril Ladder; EMMBSAR = Multidimensional Scale for the Measurement of Subjective Well-Being of Anguas-Plata and Reyes-Lagune; EWBS = Emotional Well-being Scale; GSL = Global Satisfaction with Life; GQA = General Questionnaire for Adolescents; HFS = Happiness Face Scale; HLTW = Happiness in the Last Two Weeks; HOL = Happiness Overall Life; HTOL = Happiness Taking into Account Overall Life; LS = Life Satisfaction; LSD = Life Satisfaction Domain; L3S = Life 3 Scale; OLS = Overall Life Satisfaction; MLSS = Multidimensional Life Satisfaction Scale; PANAS = Positive and Negative Affect Scale; PAS = Positive Affect Scale; PHS = Piers-Harris Children’s Concept Scale 2; PHAS = Perceived Happiness Status; PNA = Positive and Negative Affect; PNAA = Scale of Positive and Negative Affects for Adolescents; POMS = Profile of Mood States; QLQ = Quality of Life Questionnaire; RCA = Russell’s Core Affect; RGLS = Rating of Global Life Satisfaction; SLSS = Students’ Life Satisfaction Scale; SWB = Subjective Well-Being; SWLS = Satisfaction with Life Scale; WHO-5 WBI = World Health Organization-Five Well-Being Index. Family Method: I = interview assessments; P/S = parent and self-report; S = self-report. Family measures: A&B = Attitudes and Behaviors survey; AIS = Adolescent Interview Schedule; BFRS = Brief Family Relationship Scale; BMSLSS = Brief Multidimensional Students’ Life Satisfaction Scale; CSRFFI = Colorado Self-Report of Family Functioning Inventory; FACES = Family Adaptability and Cohesion Evaluation Scales; FC = Family Conflict; FCC = Family Conflict Climate; FCS = Family Conflict Scale; FCLQ = Family Closeness Questions; FCQ = Family Communication Questions; FCSFR = Family Communication Subscale of Family Relationships; FES = Family Environment Scale; FLSS = Family Life Satisfaction Scale; F/MACS = Father/Mother–Adolescent Conflict Scale; FSD = Family Satisfaction Domain; FSS = Family Satisfaction Scale; GDSI = General Domain Satisfaction Index; ISCWeB = International Survey of Children’s Well-Being; LDS = Life Domains Satisfaction; MLSSA = Family Satisfaction subscale of the Multidimensional Life Satisfaction Scale for Adolescents; MSLSS = Multidimensional Students Life Satisfaction Scale; NRI = Network of Relationship Inventory; PACS = Parent-Adolescent Communication Scale; PCI = Parent-Child Intimacy; PRI = Pictorial Representation Index; QFIS = Quality of Family Interaction Scale; QFR = Quality of Family Relationships; SDDC = Satisfaction with Different Developmental Contexts; SDLD = Satisfaction with Different Life Domains; SWF = Satisfaction with Family; SWFaL = Satisfaction with Family Life; SWFLS = Satisfaction with Family Life Scale; SWFR = Satisfaction with Family Relationships; YESIMM = Aversive Parent–Child Interactions subscale of the Youth Everyday Social Interactions and Mood Measure. Research design: C = cross-sectional study; D = derived from a longitudinal study (one wave of a longitudinal study); F = 1-year follow-up study; L = longitudinal study; V = validation study of measure. Pub = published; * = Additional data retrieved from authors. ns = not specified.

### 3.3. Global Family Functioning, Environmental Variables, and Happiness

The impact of global family functioning and family environmental variables (i.e., family relationships and family dynamics) on happiness was supported by a large number of studies (*n* = 39). Most articles (Table 2) specifically discussed the impact of dysfunctional family functioning on happiness, from both the parents’ and children’s perspectives. Many studies showed that adequate and adaptive family functioning correlated positively with higher levels of happiness [18,24,134,136,174,179,180,181,182,183,184], considering both affective and cognitive components [22,43,185]. Furthermore, some studies showed that family environment and happiness correlated with adolescents’ gender and age [46,181,186]. Only one study found no significant relation between family functioning and adolescents’ happiness [187].

Children’s and adolescents’ global happiness correlated positively with family relationships [12,90,91,188,189,190,191,192,193,194,195,196,197]. Positive relationships within the family strongly predicted increased subjective happiness [172,198,199] and low depressive symptoms. Children who reported more daily activities with family members reported higher levels of happiness, regardless of the type of activity (e.g., talking, playing, learning together). Studies also indicated that adolescents’ perceptions of high mutuality and stability and a lack of severe problems in the family predicted their global satisfaction [1,200]. Studies further suggested that perceived good relationships in the family helped adolescents to develop feelings of freedom, love, and happiness [172,194,198,199].

#### Sociodemographic Variables: Age, Gender, and Socioeconomic Status

Sociodemographic variables (e.g., age, gender, socioeconomic status) represent a subtheme of environmental factors associated with happiness (*n* = 21). The well-being of children and adolescents primarily depended on the closeness of their relationships with family members and, particularly, their parents. Children reported more satisfaction with their family relationships [198] relative to adolescents [43,146]. However, one study found no age or gender differences in the interaction between life satisfaction and family functioning [191]. Young people who perceived a higher quality parent–child relationship had greater and more stable life satisfaction from middle (i.e., aged 14–16 years) to late adolescence (i.e., aged 17–18 years) [197].

The negative correlation between family functioning and life satisfaction was affected by gender differences. Girls perceived less familial dysfunction relative to boys [46]. One study found that family satisfaction was the only significant predictor of girls’ life satisfaction [37]. Another study showed that boys with high overall satisfaction reported high stability and reciprocity and fewer problems in the family [200]. However, other studies found no gender differences in the association between these variables [136,179,201]. Only one study found no correlation between family functioning and the life satisfaction of adolescent boys from low-income families [202].

Shek (1998) [89] showed that adolescents’ life satisfaction correlated with the perceived family atmosphere (i.e., family happiness and family interactions), parent–adolescent relationship, and adolescent–parent communication at both data collection points (i.e., one year apart), regardless of gender. Thus, for both boys and girls, greater life satisfaction was associated with a higher level of perceived happiness in the family and more frequent positive conversations within the family. Some studies revealed that adolescents with a more positive family environment displayed greater happiness and life satisfaction [89,195,196]. Other studies revealed that the link between family functioning and life satisfaction was significantly stronger among adolescent girls, compared to adolescent boys [24,180].

Concerning socioeconomic status, Shek (2002) [177] showed that family functioning was more strongly related to adolescent adaptation among economically disadvantaged adolescents relative to non-economically disadvantaged adolescents. This suggests that family functioning may be associated with better adaptation in high-risk adolescents [22,161]. One study found that satisfaction with family functioning predicted the happiness of rural-urban migrant children—a subgroup with worse self-rated family financial situations [203].

**Table 2:** Sample Characteristics and Methods of Assessment of the Reviewed Studies Investigating Global Family Functioning, Environment Variables, and Happiness (*n* = 39).

	Child Characteristics			Happiness Measure		Family Measure			
Author (Year), Country	*N*	Age	% Male	Method	Measure	Method	Measure	Res. Design	Pub
Ben-Zur (2003) [12], Israel	112	Range 15–19 (*M* = 17.06)	48	S	LSS PANAS	P/S	RFMQ	C	Pub
Cacioppo et al. (2013) [136], Italy	255	Range 15–17 (*M* = 15.98)	40.8	S	MSLSS	S	FAD	C	Pub
Chui & Wong (2017) [18], China	1830	Range 10–19 (*M* = 14.2)	47.9	S	SWLS	S	FAI	C	Pub
Flouri & Buchanan (2003) [201], United Kingdom	2722	Range 14–18 (*M* = 14.2)	41.3	S	HS	S	F/MIS	C	Pub
Gilman & Huebner (2006) [188], United States	485	Range 11–18 (*M* = 14.45)	54.0	S	SLSS	S	BASC	C	Pub
Gómez et al. (2019) [1], Chile	1392	Range 10–13 (*M* = 11.5)	54.2	S	SLSS	S	ISCWeB	C	Pub
Goswami (2012) [198], United Kingdom	4673	Two age groups (8 and 10 year)	47.0	S	SLSS	S	MSLSS	C	Pub
Heaven et al. (1996) [186], Australia	183	Range 13–17 (*M* = 13.3)	36.1	S	SWLS	S	FOS	C	Pub
Huebner et al. (2000) [199], United States (Time 1)	321	Range 14–18 (*M* = 16.14)	35.0	S	SLSS	S	BASC	L	Pub
Huebner et al. (2000) [199], United States (Time 2)	99	Range 14–18	34.5	S	SLSS	S	BASC	L	Pub
Lawler et al. (2015) [189], 11 countries (United States sample)	784	Range 11–14 (*M* = 12.63)	ns	S	LSI	S	FRQ PIS	C	Pub
Lawler et al. (2015) [189], 11 countries (international sample)	781	Range 10–14 (*M* = 12.06)	ns	S	LSI	S	FRQ PIS	N	Pub
Lawler et al. (2017) [190], 11 countries (United States sample)	502	Range 10–12 (*M* = 10.66)	ns	S	LSI	S	FRQ PIS	C	Pub
Lawler et al. (2017) [190], 11 countries (international sample)	502	Range 9–12 (*M* = 10.12))	ns	S	LSI	S	FRQ PIS	N	Pub
Lawler et al. (2018) [90], South Korea and United States (SK sample)	489	Range 10–12	ns	S	SLSS	S	FRQ PIS	C	Pub
Lawler et al. (2018) [90], South Korea and United States (US sample)	1286	Range 10–12 (*M* = 11.21)	ns	S	SLSS	S	FRQ PIS	C	Pub
Nevin et al. (2005) [191], Ireland	294	Range 15–18 (*M* = 16.4)	40.0	S	OHI SWLS	S	FAD	C	Pub
Newland et al. (2014) [192], United States	149	Range 12–14 (*M* = 13.0)	52.3	S	LSI	S	FRQ PIS	C	Pub
Newland et al. (2015) [193], United States (5th grade)	502	Range 10–12 (*M* = 10.66)	54.8	S	LSI	S	FRQ PIS	C	Pub
Newland et al. (2015) [193], United States (7th grade)	784	Range 12–14 (*M* = 12.63)	49.1	S	LSI	S	FRQ PIS	C	Pub
Newland et al. (2019) [91], 14 countries	25,906	Range 9–14 (*M* = 11.4)	47.8	S	SLSS + OLS	S	FRQ	N	Pub
Rask et al. (2003) [200], Finland	239	Range 12–17 (*M* = 14.0)	49.0	S	BSW/Y	P/S	FDM II	C	Pub
Sari & Dahlia (2018) [185], Indonesia	193	Range 12–15 (*M* = 12.97)	50.3	S	SWLS PANAS	S	FAD	C	Pub
Sarriera et al. (2018) [194], Brazil and Spain	6747	Range 11–14 (*M* = 12.07)	49.3	S	SLSS	S	ISCWeB	N	Pub
Shek (1997a) [46], China	365	Range 12–16	80.5	S	SWLS	S	SFI	C	Pub
Shek (1997b) [179], China	429	Range 12–16 (*M* = 13.0)	50	S	SWLS	S	SFI	D	Pub
Shek (1998a) [180], China (Time 1)	429	Range 12–16 (*M* = 13.0)	50.6	S	SWLS	P/S	SFI	L	Pub
Shek (1998a) [180], China (Time 2)	378	Range 13–17 (*M* = 14.0)	ns	S	SWLS	P/S	SFI	L	Pub
Shek (1998c) [89], China (Time 1)	429	Range 12–16 (*M* = 13.0)	50.6	S	SWLS	S	SFI	L	Pub
						I	AIS		
Shek (1998c) [89], China (Time 2)	378	Range 13–17 (*M* = 14.0)	ns	S	SWLS	S	SFI	L	Pub
						I	AIS		
Shek (1999) [181], China (Time 1)	429	Range 12–16 (*M* = 13.0)	51.0	S	SWLS	P/S	SFI	L	Pub
Shek (1999) [181], China (Time 2)	378	Range 13–17 (*M* = 14.0)	ns	S	SWLS	P/S	SFI	L	Pub
Shek (2002b) [182], China	1519	Range 11–18	ns	S	SWLS	S	FAI	C	Pub
Shek (2002c) [134], China	361	Range 12–16 (M = 14.0)	66.4	S	SWLS	S	SFI FAD FAI	C	Pub
Shek (2002d) [177], China	229	Range 12–16	53.3	S	SWLS	S	PPAR	D	Pub
Shek (2004) [202], China	228	Range 12–16	46.5	S	SWLS	S	FAI	D	Pub
Shek (2005) [24], China (Time 1)	229	Range 12–16	46.7	S	SWLS	S	FAI	L	Pub
Shek (2005) [24], China (Time 2)	199	Range 13–17	ns	S	SWLS	S	FAI	L	Pub
Shek & Liang (2018) [43], China	3328	Range 12–18 (*M* = 12.59)	51.7	S	SWLS	S	FAI	L	Pub
Shek & Liu (2014) [22], China (Time 1)	4106	Range 14–15 (*M* = 14.65)	53.2	S	SWLS	S	FAI	L	Pub
Shek & Liu (2014) [22], China (Time 2)	2667	Range 17–18	ns	S	SWLS	S	FAI	L	Pub
Shek et al. (2001) [130], China	1519	Range 11–18 (*M* = 13.5)	49.9	S	SWLS	S	PPAR	C	Pub
Syanti & Rahmania (2019) [187], Indonesia	118	Range 12–19	44.0	S	SWBS	S	FAD	C	Un
Tang et al. (2021) [183], China	1060	Range 13–16 (*M* = 14.6)	ns	S	CHI	S	BFFQ	C	Pub *
Uusitalo-Malmivaara (2012) [195], Finland	737	Range 11–12 (*M* = 12.10)	49.2	S	SHS	S	FRS	C	Pub
Uusitalo-Malmivaara & Lehto (2013) [196], Finland	737	Range 11–12 (*M* = 12.10)	49.2	S	SHS	S	FRS	C	Pub
Wang et al. (2019) [203], China	2229	Range 9–17 (*M* = 11.46)	52.0	S	PANAS PWI-SC SWLS	S	FAPGARI	C	Pub
Willroth et al. (2021) [197], United States (Time 1)	674	Range 14–16 (*M* = 14.75)	ns	S	OLS	S	PCRQ	L	Pub
Zhou et al. (2018) [184], China	1656	Range 16–19 (*M* = 15.8)	44.39	S	HS + MSLSS	S	FAD	C	Pub

Note. Happiness method: S = self-report questionnaire. Happiness measure: BSW/Y = Berne Questionnaire of Subjective Well-Being/Youth form; CHI = Chinese Happiness Inventory; HS = Happiness Scale; LSI = Life Satisfaction Indicator; LSS = Life Satisfaction Scale; MSLSS = Multidimensional Students’ Life Satisfaction Scale; OHI = Oxford Happiness Inventory; OLS = Overall Life Satisfaction; PANAS = Positive and Negative Affect Scale; SHS = Subjective Happiness Scale; SLSS = Students’ Life Satisfaction Scale; SWBS = Subjective Well-Being Scale; SWLS = Satisfaction with Life Scale. Family Method: I = interview assessments; P/S = parent and self-report; S = self-report. Family measure: AIS = Adolescent Interview Schedule; BASC = Behavior Assessment System for Children-Self-Report-Adolescent Form; BFFQ = Brief Family Function Questionnaire; FAD = Family Assessment Device; FAI = Family Assessment Instrument; FAPGARI = Family APGAR Index; FDM II = Family Dynamics Measure; F/MIS = Father/Mother Involvement Scale; FOS = Family-of-Origin Scale; FRS = Family Relationship Scale; FRQ = Family Relationship Quality; ISCWeB = International Survey of Children’s Well-Being; MSLSS = Multidimensional Students’ Life Satisfaction Scale; PCRQ = Parent-Child Relationship Quality; PIS = Parent Involvement Scale; PPAR = Perceived Parent–Adolescent Relationship; RFMQ = Relationship with Father/Mother Questionnaire; SFI = Self-Report Family Instrument. Research design: C = cross-sectional; D = derived from a longitudinal study (one wave of a longitudinal study); L = longitudinal; N = cross-national. Publication status: Pub = published; Un = not published; * = additional data retrieved from authors. ns = not specified.

### 3.4. Parental Differences

Parent gender was a central factor in studies investigating the association between happiness and family functioning in children and adolescents (*n* = 17) (Table 3). One study revealed that perceived family competence was associated with family members’ perceptions of parental dyadic qualities and individual functioning [131]. In particular, regardless of the informant (i.e., father, mother, and child), child satisfaction correlated negatively with family dysfunction [181]. No differences emerged between parents and children regarding the impact of family conflict [129] and family satisfaction on children’s happiness [169]. Finally, one study indicated no significant differences between parents and children in the association between children’s happiness and family functioning (i.e., cohesion, adaptability, communication, and family satisfaction) [27].

While the investigated studies highlighted differences between mothers and fathers, the results were contradictory and heterogeneous. Some studies reported that maternal understanding was closely related to adolescent life satisfaction [145] and overall adolescent satisfaction [200]. Adolescents with a positive relationship with their mother showed greater happiness than those with a poor mother–child relationship; however, this association was not significant for the father–child relationship [43].

Other research found that the father–child relationship was more closely correlated with indicators of adolescents’ happiness than the mother–child relationship [12,73,129]. Furthermore, the perceived father–adolescent relationship (but not the mother–adolescent relationship) correlated positively with children’s happiness [177]. For instance, Zhao et al. (2015) showed that children’s life satisfaction correlated positively with father–child cohesion, but not mother–child cohesion [178]. Although the involvement of both the father and the mother contributed significantly and independently to children’s happiness, the involvement of the father had a more substantial effect than the involvement of the mother [201].

Children’s and adolescents’ life satisfaction was positively correlated with parent–child relationship qualities [91]. The father–adolescent relationship correlated positively with positive affect and life satisfaction, while the mother–adolescent relationship correlated positively with life satisfaction and only weakly with positive affect [12]. However, one study showed that only the perceived father–adolescent relationship correlated positively with children’s life satisfaction [177].

Age and gender differences emerged in mother–child and father–child communication. Adolescents were significantly more satisfied with their communication with their mother than their communication with their father [30]. One study showed that girls reported greater openness with their mother and boys with their father [140]. Boys reported fewer problems and more open communication with their father, relative to girls [138], while no gender differences emerged in their communication with their mother [30]. Regarding age differences, early adolescents (i.e., aged 12–13 years) reported more positive open communication with their mother and their father relative to mid-adolescents (i.e., aged 14–16 years). In addition, communication problems with both parents increased with age. Overall, adolescents were generally satisfied with their communication with their parents (particularly their mother), and early adolescents were more positive about their communication with their parents compared to mid-adolescents [30].

**Table 3:** Sample Characteristics and Methods of Assessment of the Reviewed Studies Investigating the Parental Differences (*n* = 17).

	Child Characteristics			Happiness Measure		Family Measure			
Author (Year), Country	*N*	Age	% Male	Method	Measure	Method	Measure	Res. Design	Pub
Ben-Zur (2003) [12], Israel	112	Range 15–19 (*M* = 17.06)	48.0	S	LSS PANAS	P/S	RFMQ	C	Pub
Cava et al. (2014) [140], Spain	1795	Range 11–18 (*M* = 14.2)	52.0	S	SWLS	S	PACS	C	Pub
Flouri & Buchanan (2003) [201], United Kingdom	2722	Range 14–18 (*M* = 14.2)	41.3	S	HS	S	F/MIS	C	Pub
Ingelmo & Litago (2018) [145], Spain	1409	Range 11–18 (*M* = 14.4)	49.6	S	CL	S	SWFR	C	Pub
Jackson et al. (1998) [30], Holland	660	Range 13–15 (*M* = 13.5)	46.4	S	ABS CL	S	PACS	C	Pub
Jiménez et al. (2009) [138], Spain	565	Range 11–18 (*M* = 13.6)	51.0	S	SWLS	S	PACS	C	Pub
Ljubetić & Reić Ercegovac (2020) [73], Croatia	101	Range 10–17 (*M* = 15.4)	31.7	S	GQA	S	QFIS	C	Pub
Newland et al. (2019) [91], 14 countries	25,906	Range 9–14 (*M* = 11.4)	47.8	S	SLSS + OLS	S	FRQ	N	Pub
Rask et al. (2003) [200], Finland	239	Range 12–17 (*M* = 14.0)	49.0	S	BSW/Y	P/S	FDM II	C	Pub
Schnettler et al. (2017) [169], Chile	300	Range 10–17 (*M* = 13.2)	51.0	S	SWLS	P/S	SWFaL	C	Pub
Shek (1997c) [131], China	429	Range 12–16 (*M* = 13.0)	50.6	S	SWLS	P/S	F/MACS	D	Pub
Shek (1998b) [129], China (Time 1)	429	Range 12–16 (*M* = 13.0)	50.6	S	SWLS	P/S	F/MACS	L	Pub
Shek (1998b) [129], China (Time 2)	378	Range 13–17 (*M* = 14.0)	ns	S	SWLS	P/S	F/MACS	L	Pub
Shek (1999) [181], China (Time 1)	429	Range 12–16 (*M* = 13.0)	51.0	S	SWLS	P/S	SFI	L	Pub
Shek (1999) [181], China (Time 2)	378	Range 13–17 (*M* = 14.0)	ns	S	SWLS	P/S	SFI	L	Pub
Shek (2002d) [177], China	229	Range 12–16	53.3	S	SWLS	S	F/MACS PPAR	D	Pub
Shek & Liang (2018) [43], China	3328	Range 12–18 (*M* = 12.6)	51.7	S	SWLS	S	FAI	L	Pub
Verrastro et al. (2020) [27], Italy	1549	Range 7–14 (*M* = 11.1)	47.0	G	HFS	S	FACES IV	C	Pub
				S	PHS				
Zhao et al. (2015) [178], China (father migrating group)	145	Range 10–17 (*M* = 13.9)	60.0	S	SWLS	S	FACES II	C	Pub
Zhao et al. (2015) [178], China (two-parent migrating group)	96	Range 10–17 (*M* = 13.9)	55.2	S	SWLS	S	FACES II	C	Pub

Note. Happiness method: G = graphical assessment; S = self-report questionnaire. Happiness measure: ABS = Affect Balance Scale; BSW/Y = Berne Questionnaire of Subjective Well-Being/Youth form; CL = Cantril Ladder; GQA = General Questionnaire for Adolescents; HFS = Happiness Face Scale; HS = Happiness Scale; LSS = Life Satisfaction Scale; OLS = Overall Life Satisfaction; PANAS = Positive and Negative Affect Scale; PHS = Piers-Harris Children’s Concept Scale 2; SLSS = Students’ Life Satisfaction Scale; SWLS = Satisfaction with Life Scale. Family Method: P/S = parent and self-report; S = self-report. Family measures: F/MACS = Father/Mother–Adolescent Conflict Scale; FAI = Family Assessment Instrument; FDM II = Family Dynamics Measure; F/MIS = Father/Mother Involvement Scale; FRQ = Family Relationship Quality; PACS = Parent-Adolescent Communication Scale; PPAR = Perceived Parent–Adolescent Relationship; QFIS = Quality of Family Interaction Scale; RFMQ = Relationship with Father/Mother Questionnaire; SFI = Self-Report Family Instrument; SWFaL = Satisfaction with Family Life; SWFR = Satisfaction with Family Relationships. Source of information (info). Research design: C = cross-sectional; D = derived from a longitudinal study (one wave of a longitudinal study); L = longitudinal; N = cross-national. Publication status: Pub = published. ns = not specified.

### 3.5. Longitudinal Studies and Predictions of Happiness over Time

Finally, the last theme (*n* = 13) highlighted the relevance of assessing the relation between happiness and family functioning longitudinally (Table 4). Some of the studies showed that children’s and adolescents’ life satisfaction correlated with family functioning and parental relationships over time [22,24,43,89,180,181,199]. In particular, one longitudinal study suggested that the relation between adolescents’ perceived family functioning and their psychological happiness was bidirectional [24].

Generally, the results showed that adolescent psychological happiness at Time 1 was related to perceived family functioning at Time 2. Therefore, children’s life satisfaction predicted children’s family functioning over time [181]. Moreover, the longitudinal linkage between family functioning and adolescent adjustment was stronger for adolescent girls than for adolescent boys [24]. At the same time, some studies revealed that adolescents with more poorly perceived family functioning at Time 1 (i.e., negative family environment) had poorer life satisfaction at Time 2 [22,89,180]. Notably, a negative family atmosphere, more significant family dysfunction, and more parent–adolescent conflict predicted a negative trend in adolescents’ happiness over time [89]. Overall, youth with a more positive family environment in middle adolescence (i.e., aged 14–16 years) reported higher levels of happiness during late adolescence (i.e., aged 17–18 years) [197].

Regarding the different dimensions of family functioning, studies found that family cohesion, but not perceived family adaptability, significantly predicted changes in adolescents’ happiness over time [110]. Family cohesion and open communication with parents at Time 1 positively correlated with happiness at Time 2 [175,176]. Furthermore, increased family cohesion was associated with increased life satisfaction and positive affection [110], which may have promoted happiness over time [175]. Studies also showed that parent–adolescent conflict predicted changes in adolescents’ psychological happiness over time. Thus, more significant parent–adolescent conflict at Time 1 tended to be associated with lower adolescent life satisfaction at Time 2 [89,129,181]. One study showed that children’s life satisfaction and family cohesion remained significantly related, despite gradually deteriorating during early and middle adolescence (i.e., aged 13–15 years). Youth from more cohesive families often had higher life satisfaction when they entered middle school [117], while pre-adolescents who reported higher life satisfaction at the beginning of middle school (i.e., aged 11 years) tended to experience a slower decline in family cohesion during adolescence.

**Table 4:** Sample Characteristics and Methods of Assessment of the Longitudinal Studies (*n* = 13).

	Child Characteristics			Happiness Measure		Family Measure			
Author (Year), Country	*N*	Age	% Male	Method	Measure	Method	Measure	Res. Design	Pub
Gao & Potwarka (2021) [110], China	675	Range 12–15	47.3	S	SLSS PANAS	S	FACES II	L	Pub
Huebner et al. (2000) [199], United States (Time 1)	321	Range 14–18 (*M* = 16.14)	35.0	S	SLSS	S	BASC	L	Pub
Huebner et al. (2000) [199], United States (Time 2)	99	Range 14–18	34.5	S	SLSS	S	BASC	L	Pub
Jhang (2021) [175], China (Time 1)	1273	Range 12–15 (*M* = 13.55)	49.0	S	SWLS	S	FACES III	L	Pub
Jhang (2021) [175], China (Time 2)	1028	Range 14–17	ns	S	SWLS	S	FACES III	L	Pub
Jiménez et al. (2014) [176], Spain (Time 1)	1319	Range 12–16 (*M* = 13.5)	46.0	S	SWLS	S	PACS	L	Pub
Jiménez et al. (2014) [176], Spain (Time 2)	554	Range 12–16 (*M* = 13.7)	46.0	S	SWLS	S	PACS	L	Pub
Lin & Yi (2019) [117], China	2690	Range 13–17 (*M* = 13.3)	51.2	S	LS	S	FACES III	L	Pub
Shek (1998a) [180], China (Time 1)	429	Range 12–16 (*M* = 13.0)	50.6	S	SWLS	P/S	SFI	L	Pub
Shek (1998a) [180], China (Time 2)	378	Range 13–17 (*M* = 14.0)	ns	S	SWLS	P/S	SFI	L	Pub
Shek (1998b) [129], China (Time 1)	429	Range 12–16 (*M* = 13.0)	50.6	S	SWLS	P/S	F/MACS	L	Pub
Shek (1998b) [129], China (Time 2)	378	Range 13–17 (*M* = 14.0)	ns	S	SWLS	P/S	F/MACS	L	Pub
Shek (1998c) [89], China (Time 1)	429	Range 12–16 (*M* = 13.0)	50.6	S	SWLS	S	F/MACS SFI	L	Pub
						I	AIS		
Shek (1998c) [89], China (Time 2)	378	Range 13–17 (*M* = 14.0)	ns	S	SWLS	S	F/MACS SFI	L	Pub
						I	AIS		
Shek (1999) [181], China (Time 1)	429	Range 12–16 (*M* = 13.0)	51.0	S	SWLS	P/S	SFI	L	Pub
Shek (1999) [181], China (Time 2)	378	Range 13–17 (*M* = 14.0)	ns	S	SWLS	P/S	SFI	L	Pub
Shek (2005) [24], China (Time 1)	229	Range 12–16	46.7	S	SWLS	S	FAI	L	Pub
Shek (2005) [24], China (Time 2)	199	Range 13–17	ns	S	SWLS	S	FAI	L	Pub
Shek & Liang (2018) [43], China	3328	Range 12–18 (*M* = 12.59)	51.7	S	SWLS	S	FAI	L	Pub
Shek & Liu (2014) [22], China (Time 1)	4106	Range 14–15 (*M* = 14.65)	53.2	S	SWLS	S	FAI	L	Pub
Shek & Liu (2014) [22], China (Time 2)	2667	Range 17–18	ns	S	SWLS	S	FAI	L	Pub
Willroth et al. (2021) [197], United States (Time 1)	674	Range 14–16 (*M* = 14.75)	ns	S	OLS	S	PCRQ	L	Pub

Note. Happiness method: S = self-report questionnaire. Happiness measures: LS = Life Satisfaction; OLS = Overall Life Satisfaction; PANAS = Positive and Negative Affect Scale; SLSS = Students’ Life Satisfaction Scale; SWLS = Satisfaction with Life Scale. Family Method: I = interview assessments; P/S = parent and self-report; S = self-report. Family measures: AIS = Adolescent Interview Schedule; BASC = Behavior Assessment System for Children-Self-Report-Adolescent Form; FACES = Family Adaptability and Cohesion Evaluation Scales; F/MACS = Father/Mother–Adolescent Conflict Scale; FAI = Family Assessment Instrument; PACS = Parent-Adolescent Communication Scale; PCRQ = Parent-Child Relationship Quality; SFI = Self-Report Family Instrument. Source of information (info). Research design: L = longitudinal. Publication status: Pub = published. ns = not specified.

## 4. Discussion

A total of 124 studies were systematically reviewed to identify relevant dimensions of family functioning associated with children’s and adolescents’ happiness. Four themes emerged from a review of these studies: (1) family dimensions and happiness; (2) global family functioning (i.e., family functioning and family relationships), environmental variables and happiness; (3) parental differences; (4) longitudinal studies.

Regarding the first theme, 91 studies examined the relationship between family dimensions (i.e., family cohesion and adaptability, family satisfaction and communication, and family conflict) and children’s and adolescents’ happiness. The results highlighted that family cohesion significantly predicted changes in happiness, life satisfaction, and positive affect over time [77,113,117,175]. In other words, increased family cohesion and adaptability were associated with higher levels of happiness in children and adolescents [20,110,122]. Thus, positive family dimensions may contribute directly to children’s and adolescents’ sense of happiness, contentment, and general life satisfaction [111,121].

Furthermore, in both boys and girls, positive communication with the mother and the father and high family satisfaction were directly associated with increased happiness [25,138,170,174]. The possibility to express oneself freely at home (i.e., to speak openly about any subject) was associated with greater life satisfaction for adolescents [114]. Adolescents who communicated effectively with their families probably felt that they could share their points of view and feelings openly and sincerely with their parents, and they may have interpreted this communication as a sign of parental support, trust, and closeness [30,140]. This may be especially true for girls, for whom the influence of family communication on happiness was slightly greater [27,171], possibly due to gender differences in cultural norms and socialization. Different parental socialization styles based on child gender [204] may also explain why communication tends to be more open between mothers and daughters and between fathers and sons [140].

On the other hand, communication problems and higher levels of family conflict were associated with lower happiness for children and adolescents [126,128,139]. When communication was open and trouble-free, children and adolescents were more likely to report satisfaction with their families, positive affect, and low levels of conflict, relative to children and adolescents who reported less communication with parents [30]. This finding suggests that family relationships which are perceived to be good may help children and adolescents develop feelings of freedom, love, and happiness [172], underlining that family dimensions play an essential role in influencing children’s and adolescents’ happiness [46].

As regards the second theme, 39 studies examined the association between global family functioning (i.e., family functioning and family relationships), family environment variables, and children’s and adolescents’ happiness. Specifically, a more positive perception of family functioning was related to better emotional well-being in children and adolescents [184,185,191,203]. Furthermore, regardless of the cultural background, children’s family relationships influenced their levels of happiness [1,196] more significantly than any other variable. Bad parent–child relationships were usually accompanied by lower levels of family satisfaction and happiness [145]. Thus, feeling happy at home may contribute to both boys’ and girls’ happiness [174].

The reported studies provided support for the association between global family functioning and happiness during adolescence, even though adolescents consolidate new social relationships with friends and partners during this developmental period [36]. The family is the context in which the first emotional relationships develop, and where children learn to respect and establish positive relationships of love and respect for others [194]. Parents in a well-functioning family can provide emotional support to children, allowing them to express their emotions. A warm and open family communicates happiness to children [185], giving them a sense of security, emotional connection, and trust [178].

A subtheme of environmental factors associated with happiness concerned differences in sociodemographic variables. Some family factors predicted individual differences in happiness and life satisfaction during adolescence. In particular, more positive family environments were associated with greater happiness [191,197]. Furthermore, the findings supported both stability and change in perceived levels, and the relevance of certain life satisfaction domains, among children and adolescents. Young people who perceived a higher quality parent–child relationship had elevated and stable life satisfaction from middle adolescence (i.e., aged 14–16 years) to late adolescence (i.e., aged 17–18 years) [197].

Other studies found that young people’s life satisfaction was negatively correlated with age in all global and life (i.e., family satisfaction) domains [48,146]. The decrease in happiness levels during this period suggests that pre-adolescence may be a stressful phase of development, during which cognitive, physical, and emotional changes strongly influence young people’s overall sense of happiness [27]; family members may play an essential role in accompanying them through these changes. In particular, the decline in both family cohesion and happiness during early and middle adolescence (i.e., aged 12–16 years) may be explained by both the multiple challenges that adolescents face and the more significant conflict that they tend to experience with parents, which tend to result in less participation in family activities; this may reduce adolescents’ perceived family cohesion and life satisfaction [117].

Regarding the third theme identified, 17 studies explored parental gender differences in the association between happiness and family functioning. The selected studies produced contradictory results: a single study reported that a positive mother–child relationship, but not a father–child relationship, was associated with greater happiness in children [43]. However, six studies found significant correlations with the father–child relationship and not the mother–child relationship [12,73,129,177,178,201]. These results suggest that relationships with both mothers and fathers are relevant to children’s and adolescents’ happiness.

However, the reviewed studies found that the father–child relationship was more closely related to indicators of happiness in adolescents than the mother–child relationship [12,73,129]. Indeed, the father–child relationship, father–child cohesion, and father–child conflict predicted children’s life satisfaction, while no equivalent associations were found for the mother [129,177,178]. These results suggest that the effect of father–child proximity on children’s and adolescents’ development is not related to mother–child proximity [178].

However, these studies, which suggest that fathers have the most significant impact on children’s and adolescents’ well-being, contradict the literature showing that mothers tend to be more significant in determining child developmental outcomes. While fathers tend to spend less time with children relative to mothers [205], they may be more committed and dedicated to children when they do spend time together, focusing on the specific situation at hand. Children may perceive their father’s behavior as an essential aspect of their relationship that increases their happiness over the long term [73]. Future studies should investigate the differences between mothers and fathers and the different perspectives between parents and children, to better understand these aspects.

Finally, the last theme that emerged (13 studies) highlighted the importance of evaluating the relation between happiness and family functioning over time, from a predictive perspective. Several studies showed that, regardless of the informant (i.e., father, mother, or child) and the sequence of data collection (i.e., simultaneously vs. longitudinally), children’s happiness was correlated with family functioning [89,181]. The results of both the simultaneous and longitudinal studies consistently showed that the cognitive component of happiness (i.e., life satisfaction) was significantly associated with family functioning and family relationships [22,43,199]. In addition, the longitudinal studies suggested that the relation between perceived family functioning and adolescents’ happiness may be bidirectional [24]; therefore, it is not possible to confirm a univocal causal link between these factors.

Regarding subdimensions of family functioning, studies found that family cohesion [110,175], family communication [176], and parent–adolescent conflict [89,129] significantly predicted changes in adolescent happiness over time: more significant parent–adolescent conflict at Time 1 tended to be associated with a decline in adolescent life satisfaction at Time 2 [89], and greater family cohesion and open communication with parents tended to be associated with increased life satisfaction over time [117,176]. Also, concerning family conflict, the data showed that the relation between parent–adolescent conflict and adolescent emotional well-being could be bidirectional [89]. Future studies should further investigate the causal links between individual and family variables.

In conclusion, the findings of this study suggest that family dimensions may influence the affective and cognitive components of children’s and adolescents’ happiness [30,46,77,110,111,112,124,125,135]. In particular, the reviewed findings demonstrate the significance of family bonds and support for adolescents, indicating that, when family members provide help, affection, and understanding, children and adolescents experience multiple benefits that undoubtedly affect their development of positive psychological experiences [145,200].

### Limitations and Strengths of the Studies, and Future Research Directions

Despite increasing research interest in the relation between happiness and family functioning (as evidenced by the growing number of publications in recent years), the investigated studies suffered from some methodological limitations. First, the use of self-report measures may have exposed the research to social desirability bias. Future studies should employ a multi-informant and multi-method methodology combining qualitative measures (i.e., structured or semi-structured interviews and observational measures) or multi-informant questionnaires (i.e., parent and teacher reports) with self-reports. Second, the use of cross-sectional designs did not enable causal links to be drawn between variables. Thus, future studies should implement longitudinal procedures to better understand the factors that contribute to the happiness of children and adolescents. Furthermore, the heterogeneity of the samples (with respect to, e.g., geographical scope, size, and age range) limit the generalizability of the results.

The lack of a coherent theoretical model to define the construct of happiness represents a significant gap in the literature. This may explain the variety in both measurement tools and operationalizations of the construct in the investigated studies. Compounding this, some of the investigated studies did not clearly define happiness, positive affect, or life satisfaction. Therefore, future research should explicitly make the psychological construct operational. Additionally, future research should explore the association between attachment styles and children’s and adolescents’ happiness during development.

A further limitation of the present research is the possibility that methodological biases may have affected the study selection, due to the arbitrariness of the constructs and the interpretation of the reviewers. However, two independent evaluators excluded all articles that deviated from a precise definition of happiness or that analyzed family factors other than family functioning. Thus, attempts were made to target the constructs of interest.

A future research direction might be to examine overall effect sizes, which were not addressed in the present study. Moreover, as the present work focused on the relation between happiness and family functioning in non-clinical samples, an equivalent analysis in clinical samples may provide important new insights. Finally, the present review suggests the relevance of the father–child relationship, father–child cohesion, and father–child conflict in predicting children’s and adolescents’ happiness. Future research should further investigate the differences between fathers and mothers, using multi-informant and mixed-methods procedures and a longitudinal approach.

However, the present work also has significant strengths, including compliance with a rigorous systematic review protocol with clearly-defined inclusion and exclusion criteria. Indeed, a careful research strategy carried out by two independent evaluators was employed to acquire all relevant articles. Another strength is the high reviewer reliability during the screening process, reflecting a transparent selection methodology. Uniquely, the review represents the first study to synthesize the literature on happiness in the family context during development, filling a significant gap in the literature pertaining to the possible impact of family functioning on children’s and adolescents’ happiness. Finally, the review identified heterogeneous measurements of happiness and family functioning during development, suggesting that future studies should develop a more standardized approach to obtain more consistent results.

## 5. Conclusions

The present review included studies that investigated the relationship between family functioning and happiness. The reviewed studies found a positive relation between happiness and family functioning in different cultures and age groups. Thus, family factors seem to play an essential role in increasing or diminishing the happiness of children and adolescents. However, many aspects remained largely unexplored, and more research is needed to determine how family variables (and particularly family functioning) affect children’s and adolescents’ happiness. Finally, more longitudinal studies are required to test causal relationships. Increased evidence of the potential direction of causality of these variables would extend our knowledge of happiness, as it is currently unclear whether family variables affect levels of happiness, positive affect, and life satisfaction, and whether these relationships are bidirectional. 

## Data Availability

Articles and data will be made available to the corresponding author upon reasonable request.
